# Drug Development for Alzheimer’s Disease: Microglia Induced Neuroinflammation as a Target?

**DOI:** 10.3390/ijms20030558

**Published:** 2019-01-28

**Authors:** Yuan Dong, Xiaoheng Li, Jinbo Cheng, Lin Hou

**Affiliations:** 1 Department of Biochemistry, Medical College, Qingdao University, Qingdao 266071, China; juliadong829@hotmail.com or dongyuan@qdu.edu.cn; 2 Beijing Institute for Brain Disorders, Capital Medical University, Beijing 100069, China; lxh1412@163.com; 3 The Brain Science Center, Beijing Institute of Basic Medical Sciences, 100850 Beijing, China

**Keywords:** Alzheimer’s disease, neuroinflammation, microglia, drug development

## Abstract

Alzheimer’s disease (AD) is one of the most common causes of dementia. Its pathogenesis is characterized by the aggregation of the amyloid-β (Aβ) protein in senile plaques and the hyperphosphorylated tau protein in neurofibrillary tangles in the brain. Current medications for AD can provide temporary help with the memory symptoms and other cognitive changes of patients, however, they are not able to stop or reverse the progression of AD. New medication discovery and the development of a cure for AD is urgently in need. In this review, we summarized drugs for AD treatments and their recent updates, and discussed the potential of microglia induced neuroinflammation as a target for anti-AD drug development.

## 1. Introduction

Alzheimer’s disease (AD) is a chronic neurodegenerative disorder, characterized by a gradually progressive loss of memory and cognitive functions as early symptoms, and developing into dementia eventually [1]. It is mostly diagnosed in people over 65 years-old, which is termed sporadic AD, while around 4–5% of cases occur before 65, which is classified as early-onset AD [2]. According to the recent report released by Alzheimer’s Disease International (ADI), AD has become one of the most common causes of dementia. In 2018, 50 million people are suffering from dementia, costing 1 trillion US$ globally. By 2050, the estimated number of people with dementia will reach 152 million, causing a huge social and economic burden for the families and caregivers of the patients. Incidence of AD is sex-related, which happens in women more than men [3,4]. In the United States, among the 5.5 million patients diagnosed with sporadic AD, 3.4 million are women, which makes women almost twice more vulnerable than men [5]. Multiple causes may explain this higher incidence of AD in women, including the difference of life expectancy [6], sex steroid hormones [7,8,9], and educational level [10,11] of men and women.

It has been more than a century since the first diagnosis of Alzheimer’s disease in 1906 [12], and the cause of this disease is still unclear. Consequently, pharmacological approaches to treat AD are mostly symptomatic. Currently, no drug is able to stop or reverse the progression of AD. In recent decades, amyloid-β (Aβ) plaques and tau neurofibrillary tangles aggregations have been intensively studied, and are believed to be vital targets for the cure of AD. Many new drugs have been developed and have entered clinical trials. However, up until now, no Aβ-targeting drug has been officially approved by the United States Food and Drug Administration (FDA) for the clinical treatment of AD.

Microglia-mediated neuroinflammation is one of the most remarkable hallmarks in neurodegenerative diseases. Microglia induced neuroinflammation contributes to the pathogenesis of AD by direct damage to the neuron, concurrently promoting protein aggregations, suggesting that it should be a new target for AD treatment [13]. In this review, we summarized the Aβ plaques and tau neurofibrillary tangles-targeting drugs currently undergoing clinical trials (information comes from https://clinicaltrials.gov), and discussed the potential of microglia induced neuroinflammation as a target for anti-AD drug development.

## 2. Cause of Alzheimer’s Disease

The pathology of AD includes the aggregation of extracellular senile plaques formed by Aβ protein, intracellular neurofibrillary tangles formed by hyperphosphorylated tau protein, enhanced neuroinflammation, oxidative stress, iron dysregulation, and neuronal cell death [14,15,16]. The symptoms of AD patients usually develop starting from mild cognitive impairment (MCI) at the preclinical stage, to the complete loss of language and the ability to live independently at the advanced stage. Multiple hypotheses exist trying to explain the pathogenesis of AD, including cholinergic hypothesis, amyloid cascade hypothesis, tau neurofibrillary hypothesis, mitochondrial dysfunction, and so on.

While AD is not considered a genetically inherited disease, mutations in the genes encoding the Amyloid precursor protein (APP), presenilins 1 and 2, can cause familial AD, usually with an early onset [17,18]. Apolipoprotein E (ApoE) ε4 allele is the best known genetic risk factor in the incidence of sporadic AD [1,16,19]. Individuals with ApoE ε4/ε4 genotypes have significantly increased incidences of AD compared with individuals with the ApoE ε3/ε4 genotypes [20]. Although no difference in the incidence of AD is observed between men and women of the ages between 55 to 58, women show a higher risk at an earlier age [20]. Mutations in the gene encoding the triggering receptor expressed on myeloid cells 2 (TREM2) are also proven to increase the risk of AD [21,22,23,24,25]. A TREM2 variant, rs75932628, results in an Arg47His substitution, significantly increasing the incidence of AD [21,22]. Calcium (Ca^+^), as a universal second messenger, involves in a wide range of cellular processes. Neural Ca^+^ dysfunction has been widely accepted as an important contributor in AD and other neurodegenerative diseases [26,27,28]. Functional intracellular calcium homeostasis is tightly regulated within a narrow range by Ca^+^ channels and pumps [29,30]. Calcium homeostasis modulator protein 1 (CALHM1) plays important roles in controlling the Ca^+^ influx and intracellular calcium signaling, through the activation of extracellular signal-regulated kinase-1/-2 (ERK1/2) kinase signaling cascade [31,32]. CALHM1 knocked out mice displayed an impaired memory flexibility and hippocampal long-term potentiation (LTP), indicating Ca^+^ dysregulation as an important factor in neuronal activity [32]. Other causes of AD include metal ions dysregulation and mitochondrial dysfunction related to protein aggregations, oxidative stress, and neuron death [33,34,35,36,37]. A recent meta-analysis of genome-wide association studies (GWAS) identified 19 other loci associated with AD as genetic risk factors [38].

The cholinergic hypothesis proposes that AD is caused by reduced neurotransmitter acetylcholine synthesis [39]. It is the first hypothesis established that tries to explain the onset and development of AD, and is the most important target that current clinical treatments are based on [39,40]. However, this hypothesis is still controversial, largely because of the questionable efficacy of the anti-AD drugs intended to treat acetylcholine deficiency [40].

In the amyloid cascade hypothesis, senile plaques aggregation at the extracellular region of the human brain is responsible for an amyloid neurotoxic cascade, resulting in the atrophy and degeneration in the temporal and parietal lobe, pre-frontal cortex, and hippocampus, thus causing memory and cognitive impairment, eventually developing into dementia [41,42]. Aβ protein, the core component of senile plaques, is the product of the sequential cleavage of transmembrane APP by β-secretase and γ-secretase [43,44]. In a non-amyloidogenic pathway, APP is processed by α-secretase instead of β-secretase 1 to form soluble amyloid precursor protein-α (sAPP-α), and it yields P3 as the final product [45]. Transgenic mice carrying the mutant human APP gene are able to develop AD-like symptoms, such as amyloid plaques and spatial learning deficits [46,47]. Meanwhile, ApoE enhances the break-down of the Aβ protein. The isoform ApoE ε4 is not effective in this reaction, leading to increased piled up of Aβ in the extracellular region [48]. This hypothesis is supported by the finding that 40–65% of AD patients carry at least one ApoE ε4 allele [49].

Aβ toxicity to the neuron cells and the ability to magnify its effect through positive feedback loop is the main focus of this hypothesis. Aβ accumulates at synapse, causing impairment in the synaptic functions as well as neurotransmission. Also, Aβ accumulation at the extracellular region is able to cause not only neuronal cell death, but also the loss of postsynaptic density protein 95 (PSD-95) and synaptophysin [50]. The excitotoxicity of Aβ is due to the over-excitation of *N*-methyl-d-aspartate receptors (NMDARs), and is considered as the main mechanism of Aβ-induced neuron damage [51]. Apart from that, Aβ is able to induce hyperphosphorylated tau protein accumulation, forming fibril tangles at the intracellular region [52], and causes the chronic neuroinflammation. Multiple forms of Aβ exist in the extracellular region of the AD brain. Aβ40 and Aβ42 are the most common isoforms of this protein, both of which are found in the amyloid plaques in the brains of AD patients. Aβ42 has a particularly strong tendency to aggregate, giving rise to neurotoxic components, including oligomers and plaques. In the familial AD, many genetic mutations in APP and presenilins contribute to the disease by only increasing one of the Aβ isoforms, or by just altering the Aβ40/42 ratio [53]. Furthermore, substantial evidence now indicates that soluble Aβ oligomer accumulation is corelated with the progress of AD, rather than insoluble Aβ plaques aggregation [54,55]. Apart from Aβ itself, by-products of Aβ generation may also be able to contribute to AD’s progression. The cleaved N-terminal fragment of APP (N-APP) is reported to be able to bind to death receptor 6 (DR6), and initiates the degeneration of the neuron cells [56]. The amyloid cascade hypothesis has long since been proposed, and has been intensively studied for decades [57,58]; however, it has recently been contested, mainly because of the limited outcomes of the drugs targeting it in clinical trials [59,60,61].

Another well studied hypothesis is the Tau hypothesis, which proposes that the hyperphosphorylated tau protein aggregates to form neurofibrillary tangles at the intracellular space, which contributes to the cascade of the disease [62]. Tau protein is the stabilizer of microtubules, and it plays vital roles in the cell transport system, thus its abnormality affects the functions and synaptic transmission of AD [63]. Of more importance, the hyperphosphorylation of tau protein causes the collapse of microtubules, leads to a cascade disintegration, and eventually destroys the fundamental structure of the neuron’s transport system, which end ups with neuronal cell death [64,65].

## 3. Microglia Induced Neuroinflammation in Alzheimer’s Disease

Microglial cells are located throughout the central nerve system (CNS), and account for around 15% of the total cellular population in the brain [66]. Functioning as the resident macrophages, they are critical components in the immune defense and homeostasis maintenance of the CNS [67,68,69]. Microglia scavenges throughout the brain, activated by the presence of pathogenic invasion, tissue injury, and protein aggregates, and through receptors, it recognizes danger-associated molecular patterns (DAMPs) or pathogen-associated molecular patterns (PAMPs) [68,69,70]. Upon activation, microglia is able to migrate to the site of injury and initiate an innate immune response. Simultaneously, microglia is an important component in the protection and remodeling of synapse plasticity, which is important for the normal functions of neuronal circuits [71]. Furthermore, microglia plays important roles in learning and memory functions, through the protection and remodeling of learning-related synapse [72]. Using single-cell RNA sequencing, a novel subtype of microglia, disease-associated microglia (DAM), associated with the AD progression in both mouse models and human patients, is discovered [73], indicating the vital roles of the microglia and its dynamic in the progression of AD. Generally, DAM displays a unique two-step activation mechanism along with the progression of AD; it starts with an initial TREM2-independent activation, which involves changes in the microglia markers and genes associated with AD, followed by a second TREM2-dependent activation characterized by expressing high levels of lipid metabolism and phagocytic genes signatures [73]. As the most important factor correlated with AD, aging, especially the aging of the microglia, is a vital factor in the progression of AD. Aged microglia expresses high levels of pro-inflammatory cytokines, including interleukin-1beta (IL-1β), tumor necrosis factor-alpha (TNF-α), and IL-6 [74], and undergoes a series of changes in the phenotypes and functions [75]. Furthermore, dystrophic microglia are both found in both the aged and AD brain [76]. Recent research reveals a direct link between inflammasome activation and age-related functional decline [77], meanwhile, neuroinflammation induced by microglia has been proofed to be a vital factor in the progression of AD, suggesting the close relationship among immune-aging, neuroinflammation, and AD.

**Figure 1:**
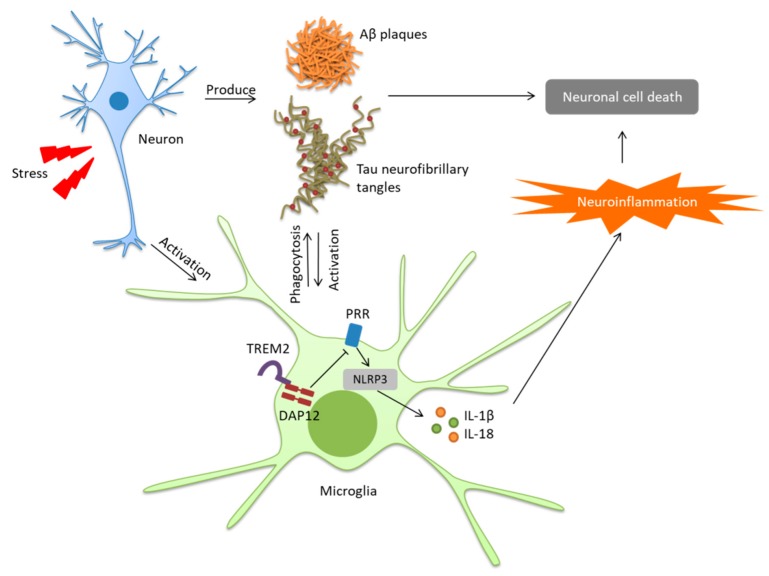
Microglia induced neuroinflammation in Alzheimer’s disease. Under the pathology of Alzheimer’s disease (AD), the accumulation of amyloid-β (Aβ) plagues and tau neurofibrillary tangles induce microglial M1-like activation, which produce inflammatory cytokines and cause neuronal cell death. Meanwhile, M2-like microglia is able to reduce Aβ plagues and tau neurofibrillary tangles accumulation by phagocytosis.

Neuroinflammation usually refers to a CNS specific, noninfectious chronic inflammation-like glial response that leads to neuronal degradation [78]. Microglia participates in the neuroinflammation through a series of intercellular communications. Reactive microglia release proinflammatory cytokines such as interleukin-1beta (IL-1β), IL-6, IL-18, and tumor necrosis factor-alpha (TNF-α), and up-regulates the expression of chemokines such as CCL2, CCR3, and CCR5, resulting in local inflammatory responses, causing the death of neural cells [79,80,81,82]. In AD, the microglia is able to contribute to the neuroinflammation under the stimulation of Aβ oligomers and plagues via cell surface receptors, including toll-liker receptors (TLRs); LPS receptor cluster of differentiation 14 (CD14); and scavenger receptors such as SCARA1, CD36, and CD47 [83,84,85,86,87]. CD36 is able to recognize and bind with Aβ, causing the activation of the NACHT, LRR, and PYD domains-containing protein 3 (NLRP3) inflammasome, which then activates proinflammatory cytokines IL-1β (Figure 1). Meanwhile, the knockout of CD36 in the macrophages prevents the activation of NLRP3 inflammasome, IL-1β release, and intracellular Aβ accumulation [88]. NLRP3 belongs to the NOD-like receptor superfamily, mainly expressed in the microglia in the brain, functioning as a pattern recognition receptor (PRR) in the innate immune system. NLRP3 inflammasome is activated in AD [89,90]. The deletion of either NLRP3 or downstream regulator caspase-1 is able to rescue the symptoms of AD in APP/SP1 mice [90]. Furthermore, the association of immune receptors, including TREM2 and CD33 with Alzheimer’s disease, indicate the important part neuroinflammation played in this disease [22,91,92,93]. In sporadic AD, reduced Aβ clearance can be linked with insufficient microglial phagocytic capacity, which is characterized by the down-regulation of Aβ phagocytosis receptors and increased cytokine concentrations [94,95].

Microglia activation can be either beneficial or detrimental in the pathogenesis of AD. Research indicates that early activations of microglia in AD present neuroprotective functions by promoting Aβ clearance, however, in response to Aβ aggregation along with the progress of the disease, proinflammatory cytokines production down-regulates the expression of Aβ clearance-related components, and hence, in turn, promotes the Aβ aggregation and neurodegeneration [94]. 

Peripheral macrophages present a diverse range of phenotypic states, from the proinflammatory M1 phenotype to the alternative activation M2 phenotype, especially under chronic inflammation conditions [96]. Likewise, microglia also presents similar polarizations during chronic inflammation, although the exact classifications are still under debate [97,98]. Generally, the LPS-induced M1-like phenotype of the reactive microglia releases proinflammatory cytokines, such as IL-1β, IL-6, IL-18, TNF-α, and ROS, in order to fight against pathogens invasion and the tumor cell; meanwhile, the M2-like phenotype of microglia produces predominantly anti-inflammatory cytokines, such as IL-10, IL-4, IL-13, and TGF-β [97,98]. In vivo study has revealed that the presence of Aβ is able to induce the phagocytosis of microglia [99], which indicates the protective function of reactive microglia against Aβ accumulation. However, in vitro evidence suggests that the ability of the phagocytosis of microglia is inhibited in AD [100]. LPS induced M1-like reactive microglia present significantly reduced activity in the phagocytosis of Aβ, while this reduction can be rescued by IL-4 induced M2-like microglia activation [100]. Moreover, proinflammatory cytokines TNF-α and IFNγ are able to inhibit Aβ uptake and internalized degradation, while anti-inflammatory cytokines IL-10 promote this ability [101,102]. This phenomenon give rise to a hypothesis that indicates that Aβ accumulation in AD may be due to the alternation of the microglia phenotype. Therefore, the modulation of microglia towards M2-like reactive microglia may have its potential benefit in AD treatment (i.e., the conversion of reactive microglia from a detrimental to beneficial factor). Recent research suggests that the inhibition of NLRP3 inflammasome using a new drug, MCC950, or its downstream effector caspase-1, is able to increase the clearance of Aβ and improve the cognitive function in APP/PS1 mice [103,104]. Together with the evidence indicating that the polymorphisms of NLRP3 are related to the incidence of sporadic AD, this suggests an important role of NLRP3 inflammasome in converting microglia for beneficial effects, and as a promising new target for AD treatments [105,106]. 

## 4. Drugs for Alzheimer’s Disease Treatment

### 4.1. Current Drugs

The drugs currently used for AD can only temporarily relieve its symptoms; meanwhile, no medication is able to stop or reverse the underling progress of this disease. The loss of neurons and synapse in the brain is considered the most direct causes of symptoms in AD. Currently, only five drugs have been approved by the FDA for clinical treatments of AD. Four drugs, tacrine, rivastigmine, galantamine, and donepezil, are acetylcholinesterase inhibitors (AChEIs), thereby enhancing the concentration and duration of the action of the neurotransmitter acetylcholine (Ach). Tacrine is the first approved drug for mild to moderate AD [107]. Because of its safety issue on hepatotoxicity and its uncertain efficacy, it was discontinued in the USA in 2013 [108,109,110,111]. Rivastigmine and galantamine are proved to have a beneficial effect in the treatment of mild to moderate dementia of the Alzheimer’s type [112,113,114,115], while donepezil is used for the treatment of mild, moderate, and severe dementia in AD [116,117]. AChEIs are used to improve the cognition and behavior of AD patients, but are not able to slow down the progression of or reverse the disease; meanwhile, the use of these drugs is usually accompanied with common adverse effects such as nausea, vomiting, diarrhea, headaches, and dizziness [118,119,120]. Memantine, another clinically used drug is an *N*-Methyl-d-aspartate (NMDA) receptor antagonist. In the brain of AD patients, NMDA receptors are over simulated because of the overload release of glutamate by neurons, causing an increased level of calcium influx and neuronal cell death. Memantine displays some beneficial effects on the symptoms in moderate-to-severe Alzheimer’s disease, whereas it displays limited effectiveness in the mild disease [121,122].

### 4.2. Drugs Targeting Amyloidogenic Route

As a key component in the pathogenesis of AD, extracellular Aβ aggregation is one of the most studied targets for drug development. Strategies mostly include the prevention of Aβ production and aggregation, as well as anti-Aβ vaccines for immunotherapies. Drugs targeting Aβ is one of the most studied areas in AD. Generally, the strategies for drug development include the reduction of Aβ production or aggregation, and the promotion of Aβ clearance (Table 1). In order to reduce the production of Aβ, three enzymes are usually targeted, namely: α-secretase, β-secretase, and γ-secretase. This can be approached by enhancing the activity of α-secretase or by suppressing the β- and γ-secretase.

#### 4.2.1. α-Secretase Enhancer

Acitretin is a retinoid drug that has been wildly used in the treatment of dermatologic diseases such as psoriasis and hidradenitis suppurativa (HS) [123,124,125], which has recently been reported to act as α-secretase enhancer promoting the non-amyloidogenic APP pathway in patients with mild to moderate AD [126]. Its phase II clinical trials (NCT01078168) have recently been completed, showing a significant increase in the cerebrospinal fluid (CSF) soluble alpha-cleaved amyloid precursor protein (APPsα) concentration in patients receiving oral treatment of acitretin over patients receiving the placebo. Epigallocatechin-gallate (EGCG) is a green tea polyphenol that is suggested to have neuroprotective properties in neurodegenerative diseases [127,128], and has completed phase II and III clinical trials (NCT00951834) for early stage AD. Etazolate (EHT0202) as a γ-aminobutyric acid (GABA)_A_ receptor modulator and α-secretase activator is proven to present neuroprotective functions [129,130]. Its phase II clinical trial in AD treatment (NCT00880412) was completed in 2009, although no results or further studies have been published since then. 

#### 4.2.2. β-Secretase Inhibitor

Lanabecestat (also referred as AZD3293 and LY3314814) is a β-secretase inhibitor with previous studies indicating the long time reduction of plasma Aβ in AD patients [131,132]. Multiple phase II and III clinical trials have been conducted for early to mild AD treatment, however they were later terminated because of a lack of effect (NCT02245737, NCT02972658, and NCT02783573) [133]. LY3202626 is a β-secretase inhibitor that was in phase II clinical trials, which were terminated recently because of a small probability of achieving significant results (NCT02791191). LY2886721 was terminated in the phase I/II trials because of abnormal liver biochemical tests in some of the participants (NCT01561430). Verubecestat (MK-8931) is a β-secretase inhibitor reported to reduce CNS Aβ in both animal models and AD patients [134,135,136,137]. However, two clinical trials of this drug were terminated recently (NCT01739348 and NCT01953601). Multiple failures in β-secretase inhibitors for AD treatment led to a change of strategy to target the patients in the early stages of AD, or individuals with a higher risk [133]. Elenbecestat (E2609) is a small molecule β-secretase inhibitor with two phase III clinical trials (NCT02956486 and NCT03036280) currently ongoing for efficacy and safety evaluation in early AD treatment, and one phase II trial (NCT02322021) for MCI and mild to moderate AD. Atabecestat (JNJ-54861911) is a β-secretase inhibitor proven to reduce CSF-Aβ concentrations in a dose-dependent manner in both healthy elderlies and early stage AD patients [138,139,140]. Two phase II/III clinical trials of this drug are still ongoing (NCT01760005 and NCT02569398). However, an extension study for its long-term safety and tolerability was terminated recently because of benefit risk (NCT02406027). CNP520 is a β-secretase inhibitor developed for AD patients in the very early stages [141,142]. Prevention trials of CNP520 in healthy adults over 60 years-old and individuals carrying the APOE ε4 allele show a significant dose-dependent reduction in the CSF-Aβ concentrations [141,142]. Two phase II/III clinical trials of this drug in healthy participants at risk of onset of AD (60–70 years-old and APOE ε4 allele carriers) are ongoing (NCT03131453 and NCT02565511).

#### 4.2.3. γ-Secretase Inhibitor

Gamma-secretase inhibitor is another approach to reduce Aβ production. Semagacestat is the first γ-secretase inhibitor that has entered the phase III clinical trials. It is reported to have dose-dependent cognitive and functional worsening, which led to the termination of its tests. Other adverse effects include weight loss, risk of skin cancer, and infection (NCT01035138, NCT00762411, and NCT00594568) [143]. Another γ-secretase inhibitor, Avagacestat, was discontinued because of a lack of efficacy [143,144,145,146].

#### 4.2.4. Aβ Aggregation Inhibitor

Another approach targeting the amyloidogenic route is inhibiting Aβ aggregation (Table 1). PBT2 is a metal protein-attenuating compound (MPAC), however its phase II/III clinical trial was announced as failed because of a lack of efficacy [143,147]. Scyllo-inositol is an inositol stereoisomer and Aβ aggregation inhibitor. Its phase II clinical trial demonstrated some beneficial effects in mild to moderate AD, however was insufficient for a robust benefit conclusion because of its small sample size [148,149]. Tramiprosate (homotaurine, 3APS) is an amino acid originally found in seaweed, with homology with taurine and 4-aminobutyrate (γ-aminobutyric acid—GABA), act as a GABA receptor agonist, and therefore demonstrates neuroprotective effects [150]. Most importantly, tramiprosate is able to inhibit Aβ aggregation by binding to Aβ and preventing β-sheet formation [150]. In vitro and in vivo studies have revealed that tramiprosate is able to attenuate the long-term potentiation (LTP) inhibition cause by Aβ toxicity, and dose-dependently reduce soluble and insoluble Aβ in transgenic mice [151,152]. However, its phase III clinical trials have been marked as unknown, with the last update date back in 2007 (NCT00314912, NCT00217763, and NCT00088673). Later, in 2009, a publication revealed that tramiprosate treatments in mild to moderate AD patients showed some beneficial effects, but did not reach statistical significance [153]. Recent studies of tramiprosate demonstrated the clinical benefits of this drug in ApoE ε4/ε4 patients [154,155,156,157]. New drugs developed based on tramiprosate—including ALZ-801, a prodrug of tramiprosate, and GQD-T, the combination of graphene quantum dots (GQDs) and tramiprosate—have both demonstrated promising results in AD treatment [158,159,160]. Sodium oligo-mannurarate (GV-971) is an Aβ aggregation inhibitor [161], and recently completed a 36-week phase III clinical trial for the treatment of mild to moderate AD (NCT02293915). A new phase I clinical trial of GV-971 is currently investigating the safety and pharmacokinetics studies (NCT03715114)

#### 4.2.5. Aβ Vaccines

Immunotherapies targeting Aβ include vaccines and antibodies. A first generation vaccine, AN-1792, was terminated because of the incidence of adverse effects such as cerebral inflammation (NCT00021723) [162]. A follow-up study of the long term effect of this drug in AD revealed a significantly lower Aβ volume in the brain of AD patients, however no significant improvement in the symptoms of dementia [163]. Followed by this, new vaccines, CAD 106 and Vanutide cridificar (ACC-001), recently finish phase II clinical trials, with promising results [143,164,165,166]. A phase II/III clinical trial of CAD106 is still ongoing (NCT02565511).

#### 4.2.6. Aβ Antibodies

Anti-Aβ antibodies are termed as passive immunotherapy. Bapineuzumab and solanezumab are monoclonal antibodies against Aβ (1–6) and Aβ (12–28), respectively [167,168]. In 2012, a clinical trial of bapineuzumab was terminated at phase III. Its treatment in AD patients displayed a significant decrease in the senile plaques and tau protein in CSF, meanwhile, showed no improvement in the cognitive functions [143]. Three phase III trials of solanezumab were recently terminated because of a lack of efficiency in prodromal and mild AD (NCT01127633, NCT01900665, and NCT02760602). Two other phase II/III clinical trials for old individuals with at risk of memory loss and familial AD are currently active (NCT02008357 and NCT01760005). Ponezumab is a human monoclonal antibody against Aβ40. Ponezumab administration demonstrates a dose-dependent increase in plasma Aβ40 and a decrease in the hippocampal Aβ concentration in transgenic mice [169]. Its phase II trials were completed with results indicating a dose-dependentl increase of plasma Aβ, with no effect in the CSF biomarkers, brain Aβ load, and cognitive improvements [170,171,172]. GSK933776 produced promising results in phase I clinical trials [173,174], however no new clinical trial has been conducted since 2013. LY2599666 is an Aβ antibody for MCI or AD, and was terminated in a phase I trial because of a lack of efficacy (NCT02614131). Octagam^®^ 10% (IVIG) is an immune globulin intravenous (10% solution). Two phase III clinical trials of Octagam^®^ 10% for AD were terminated because of a lack of efficacy (NCT01736579 and NCT01524887), while other phase II/III trials for AD and mild cognitive impairment (MCI) are currently active (NCT01561053, NCT01300728, and NCT03319810). Aducanumab (BIIB037) is a human monoclonal antibody against aggregated forms of Aβ, including soluble Aβ oligomer and insoluble Aβ fibrils [175,176]. Aducanumab treatments have demonstrated beneficial effects in Aβ plaque clearing and a reverse calcium dysfunction in a Tg2576 mice model of AD [177,178]. In an ongoing phase I clinical trial, aducanumab treatment is able to reduce Aβ plaques and attenuate the decline of cognitive functions of prodromal or mild AD patients (NCT01677572) [179,180,181]. Two phase III clinical trials of aducanumab in early AD, and two trials for MCI or mild AD are currently ongoing (NCT02484547, NCT02477800, NCT03639987, and NCT01677572). Crenezumab is a monoclonal IgG4 Aβ antibody able to recognize multiple forms of Aβ, especially oligomers, and inhibit Aβ aggregation [182]. Although previous clinical trials of this drug failed to significantly improve the symptoms of mild or moderate AD, some beneficial effects with high dose crenezumab indicated potential treatment effects [183,184]. Multiple clinical trials of crenezumab are currently ongoing (NCT03491150, NCT03114657, NCT02670083, NCT01998841, and NCT02353598). Gantenerumab is a fully human IgG1 Aβ antibody that recognizes a conformational epitope of Aβ fibrils in the sub-nanomolar affinity in vitro, and therefore can bind to the aggregated Aβ with a high affinity [185]. The experimental results demonstrate that gantenerumab is able to enhance the phagocytosis of Aβ in brain slices co-cultured with macrophages, neutralize the inhibitory effects on LTP caused by Aβ42 oligomers in rat brains, promote cerebral Aβ clearance by recruiting microglia in a transgenic mouse model of AD, and therefore inhibit the formation of Aβ plaque [185]. Clinical trials of this drug have come out showing a reduction in the cerebral Aβ in AD patients [186,187,188]. Recently, a phase III clinical trial of gantenerumab in prodromal AD was stopped because of a lack of effects, however some dose-dependent beneficial effects indicated a probability of reaching significance with higher doses [189]. Four phase III clinical trials of gantenerumab are active for prodromal to mild AD (NCT03443973, NCT03444870, NCT02051608, and NCT01224106). The monoclonal antibody for Aβ fibrillars SAR228810 is a next generation product of murine antibody SAR255952, and has completed a phase I clinical trial with no further clinical studies ongoing. 

### 4.3. Drugs Targeting Tau Protein

Currently, several drugs targeting tau protein have entered clinical trials (Table 1). Aggregation inhibitors, TRx0014 and LMTM (TRx0237), are methylene blue dye derivatives that are able to prevent tau and amyloid aggregation [190]. LMTM is the next generation drug of TRx0014, which has produced promising results in clinical trials [143]. However, in recent trials, LMTM demonstrated no significant beneficial effect in the add-on therapy of mild to moderate AD (NCT01689246) [191]. Meanwhile, another phase III clinical trial (NCT01689233) for LMTM as a monotherapy for mild to moderate AD demonstrated promising results, indicating that LMTM might be effective in future studies [192]. New phase II/III clinical trials of LMTM for early AD (NCT03446001) and mild to moderate AD (NCT03539380) have recently started. Tideglusib is a GSK3-β inhibitor able to prevent tau hyperphosphorylation, however phase II clinical trials of this drug display no significant efficacy to AD [193].

Human tau antibody ABBV-8E12 is safe to use and is currently in phase II clinical trials for early AD (NCT02880956 and NCT03712787) [194]. RO7105705 is an antibody targeting extracellular tau, therefore stopping the spread of pathological tau, demonstrating effective outcomes in preventing tau pathology in tau-P301 transgenic mice, and attenuating microglia induced inflammation [195]. One phase II clinical trial of RO7105705 for prodromal to mild AD is ongoing (NCT03289143). AADvac1 is the first human pathologically modified tau vaccine for active immunotherapy, able to reduce 95% of the hyperphosphorylation of tau and improve the symptoms of transgenic mice [196]. Currently, early phase clinical trials have been completed with promising results [197,198]; a phase II clinical trial is ongoing for mild AD (NCT02579252). TPI 287 is a microtubule stabilizer that able to bind on tubulin and stabilize the microtubule [199], and is currently in a phase I clinical trial (NCT01966666).

### 4.4. Drugs Targeting Inflammation

#### 4.4.1. Non-Steroidal Anti-Inflammatory Drugs

The potential beneficial effect in the non-steroidal anti-inflammatory drugs (NSAIDs) has drawn public attention because several systematic reviews revealed that long term users of NSAIDs showed lower risks in the incidence of AD [200,201,202,203]. In a recent study, the NSAIDs of the fenamate class displayed a significant effect in inhibiting the NLRP3 inflammasome activation in vitro, and attenuated microglia activation in transgenic mice [204]. However, randomized controlled trials (RCT) for relationships of NSAIDs usage and AD risk failed to show significance among the normal population without dementia or AD patients [205,206,207], leaving the effectiveness of NSAIDs in AD treatment controversial.

Ibuprofen is one of the most used NSAIDs reported to have a protective effect against incidence of AD, along with other NSAIDs [202]; later, RCTs proved it to have no effect on cognitive progresses in AD [207,208]. One clinical trial of ibuprofen in AD was marked as unknown (NCT00239746), with no update since 2009. A recent phase III clinical trial of a combination treatment of ibuprofen and cromolyn (ALZT-OP1) in early AD is ongoing (NCT02547818). Tarenflurbil is a NSAID structurally related to ibuprofen, but its poor ability in brain penetration and its lack of efficacy for AD treatment has led to failure in phase III clinical trials [143,209]. Salsalate is a NSAID that is currently in an ongoing phase I clinical trial in patients with mild to moderate AD (NCT03277573). Celecoxib is also a NSAID whose phase III clinical trials for AD treatment were completed in 2016 (NCT00007189)

#### 4.4.2. Other Drugs Targeting Inflammation

Resveratrol is an antioxidant that recently completed a phase II clinical trial for the treatment of AD. As a result, resveratrol was found to be safe and well-tolerated, with the ability to cross the blood–brain barrier, although further study is required in order to determine its efficacy in AD treatment [210]. Etanercept is a TNF-α inhibitor. The phase II study of its safety and tolerability of this drug was conducted recently, and showed positive results [211]. Simvastatin is a cholesterol targeting 3-hydroxy-3-methyl-glutaryl-coenzyme A reductase (HMG-CoA reductase) inhibitor. Previous studies indicated that despite a significant decrease in the cholesterol level, no marked beneficial effect is associated with the use of this drug [212]. Neflamapimod (VX-745) is a selective inhibitor of the α isoform of the mitogen-activated serine/threonine protein kinase p38 MAPK (p38 MAPKα), and is reported to be able to slow the progression of transgenic AD mice [213]. A phase II clinical trial of neflamapimod demonstrated improvement in the episodic memory of AD patients [214]. Two phase II clinical trials of this drug in the inflammation in AD are currently ongoing (NCT03435861 and NCT03402659). Azeliragon (TTP488) is a small molecule inhibitor of the receptor for advanced glycation endproducts (RAGE) and has been reported to slow cognitive decline in AD patients [215,216], however two phase III clinical trials were terminated recently because of a lack of efficacy (NCT02080364 and NCT02916056). Pioglitazone is a peroxisome-proliferator activated receptor γ (PPARγ) agonist that showed promising results in AD treatment, however two phase III trials were terminated recently because of a lack of efficacy (NCT01931566 and NCT02284906) [217,218,219].

## 5. Conclusive Remarks

Alzheimer’s disease has become an important disease affecting the healthy aging of humans. AD is caused by multiple factors and cannot be explained by a solo hypothesis. Currently, 2103 studies can be found on the clinicaltrials.gov website under the category of Alzheimer’s disease, including drugs, therapies, and imaging markers. Drugs targeting different aspects of this disease have been intensively studied, however no effective drug has been developed for clinical use; meanwhile, many promising drug developments have been terminated at late clinical trials. For the recent decades, Aβ and tau accumulation in the brain, as the most used hallmarks of AD, have been intensively studied as drug targets for a promising cure for this disease. However, to date, drugs targeting Aβ or tau are able to present only limited beneficial effects on the pathogenesis of AD, which results in the concern of strategies for adjustment in drug development in AD. Recent advances in the understanding of the important role played by chronic neuroinflammation induced by microglia in AD, indicates a potential target for AD treatment.

## Figures and Tables

**Table 1:** Drug development for Alzheimer’s disease (AD).

Drug	Description	Phase	CT Identifier	Status
**Drugs Target Amyloid β Production**				
Acitretin	α-secretase enhancer	II	NCT01078168	Completed
Epigallocatechin-Gallate (EGCG)	α-secretase enhancer, prevent amyloid-β (Aβ) aggregation	II/III	NCT00951834	Completed
Etazolate (EHT-0202)	γ-aminobutyric acid GABA)_A_ receptor modulator, α-secretase enhancer	II	NCT00880412	Completed
Lanabecestat (AZD3293, LY3314814)	β-secretase inhibitor	II/III	NCT02245737 NCT02972658 NCT02783573	Terminated
LY3202626	β-secretase inhibitor	II	NCT02791191	Terminated
LY2286721	β-secretase inhibitor	I/II	NCT01561430	Terminated
Verubecestat (MK-8931)	β-secretase inhibitor	II/III	NCT01739348 NCT01953601	Terminated
Atabecestat (JNJ-54861911)	β-secretase inhibitor	II/III	NCT02569398 NCT01760005	Active, not recruiting
		II/III	NCT02406027	Terminated
Elenbecestat (E2609)	β-secretase inhibitor	III	NCT03036280 NCT02956486	Recruiting
		II	NCT02322021	Active, not recruiting
CNP520	β-secretase inhibitor	II	NCT02565511 NCT03131453	Recruiting
Semagacestat	γ-secretase inhibitor	III	NCT01035138 NCT00762411 NCT00594568	Completed
Avagacestat (BMS-708163)	γ-secretase inhibitor	II	NCT00890890 etc.	Terminated
**Drugs prevent Amyloid β Aggregation**				
PBT2	metal protein-attenuating compound (MPAC), Aβ aggregation inhibitor	II/III	NCT00471211	Terminated
Scyllo-inositol (ELND005, AZD-103)	inositol stereoisomer, Aβ aggregation inhibitor	II	NCT00934050, NCT00568776, NCT01735630	Completed
Tramiprosate (3APS)	Prevent β-sheet formation, Aβ aggregation inhibitor	III	NCT00314912, NCT00088673, NCT00217763	Unknown
GV-971	Aβ aggregation inhibitor	III	NCT02293915	Completed
**Immunotherapy**				
AN-1792 (AIP-001)	Anti-Aβ vaccine	II	NCT00021723	Terminated
CAD106	Anti-Aβ vaccine, induce Anti-Aβ antibody	II	NCT02565511	Recruiting
Vanutide cridificar (ACC-001)	Anti-Aβ vaccine	II	NCT00960531, etc.	Terminated
Bapineuzumab (AAB-001)	Anti-Aβ monoclonal antibody	III	NCT00676143, etc.	Terminated
Solanezumab (LY2062430)	Anti-Aβ IgG1 monoclonal antibody	III	NCT01127633 NCT01900665 NCT02760602	Terminated
		II/III	NCT02008357 NCT01760005	Active, not recruiting
Ponezumab (PF-04360365)	Anti-Aβ IgG2 antibody	II	NCT00722046, NCT00945672	Completed
GSK933776	Anti-Aβ antibody	I	NCT00459550, NCT01424436	Completed,
LY2599666	Aβ antibody	I	NCT02614131	Terminated
Octagam^®^ 10%	Immune globulin intravenous, 10% solution	III	NCT01736579 NCT01524887	Terminated
		II/III	NCT01561053 NCT01300728	Active, not recruiting
		II	NCT03319810	Enrolling by invitation
Aducanumab (BIIB037)	Anti-Aβ IgG1 monoclonal antibody	III	NCT02484547 NCT02477800	Active, not recruiting
		II	NCT03639987	Recruiting
		I	NCT01677572	Active, not recruiting
Crenezumab (MABT5102A, RG7412)	Anti-Aβ IgG4 antibody	I/II/III	NCT02670083 NCT01998841 NCT02353598	Active, not recruiting
		III	NCT03491150 NCT03114657	Recruiting
Gantenerumab (R1450)	Anti-Aβ IgG1 antibody	III	NCT01224106 NCT02051608	Active, not recruiting
		III	NCT03444870 NCT03443973	Recruiting
		II/III	NCT01760005	Active, not recruiting
SAR228810	Anti-Aβ monoclonal antibody	I	NCT01485302	Completed
**Drugs Target Tau Production**				
TRx0014	Methylene blue, tau aggregation inhibitor	II	NCT00684944 NCT00515333	Completed
LMTM (TRx0237)	Methylene blue, tau aggregation inhibitor	II/III	NCT03446001	Recruiting
			NCT03539380	Available
		III	NCT01689246 NCT01689233	Completed
Tideglusib (NP031112)	GSK3-β inhibitor, prevent tau hyperphosphorylation	I/II	NCT00948259 NCT01350362	Completed
ABBV-8E12	Anti-tau antibody	II	NCT02880956	Recruiting
		II	NCT03712787	Not yet recruiting
RO 7105705	Anti-tau antibody	I	NCT02820896	Completed
		II	NCT03289143	Recruiting
AADvac1	Tau vaccine	I	NCT02031198 NCT01850238	Completed
		II	NCT02579252	Active, not recruiting
TPI 287	abeo-taxane, bind on tubulin, and stabilize microtubule	I	NCT01966666	Active, not recruiting
**Drugs Target Inflammation**				
Ibuprofen	non-steroidal anti-inflammatory drugs (NSAIDs)	III	NCT02547818	Recruiting
Tarenflurbil	NSAIDs	III	NCT00380276 NCT00322036	Terminated
Salsalate	NSAIDs	I	NCT03277573	Recruiting
Celecoxib	NSAIDs	III	NCT00007189	Completed
Resveratrol	Phenol, antioxidant	II	NCT01504854 NCT01716637 NCT00678431	Completed
Etanercept	Tumor necrosis factor-alpha (TNF-α) inhibitor	I/II	NCT01068353 NCT01716637	Completed
Simvastatin	3-hydroxy-3-methyl-glutaryl-coenzyme A (HMG-CoA) reductase inhibitor, cholesterol targeting	II	NCT00939822	Active, not recruiting
Neflamapimod (VX-745)	p38 mitogen-activated serine/threonine protein kinase p38 MAPK (p38 MAPKα) selective inhibitor	II	NCT03435861 NCT03402659	Recruiting
Azeliragon (TTP488)	Receptor for advanced glycation endproducts (RAGE) inhibitor	III	NCT02080364 NCT02916056	Terminated
Pioglitazone	Peroxisome-proliferator activated receptor γ (PPARγ) agonists	III	NCT01931566 NCT02284906	Terminated
